# 3D Organoids: An Untapped Platform for Studying Host–Microbiome Interactions in Esophageal Cancers

**DOI:** 10.3390/microorganisms9112182

**Published:** 2021-10-20

**Authors:** Samuel Flashner, Kelley S. Yan, Hiroshi Nakagawa

**Affiliations:** 1 Herbert Irving Comprehensive Cancer Center, Columbia University Irving Medical Center, New York, NY 10032, USA; sf3070@cumc.columbia.edu (S.F.); ky2004@cumc.columbia.edu (K.S.Y.); 2 Division of Digestive and Liver Diseases, Department of Medicine, Columbia University Irving Medical Center, New York, NY 10032, USA; 3 Department of Genetics and Development, Columbia University Irving Medical Center, New York, NY 10032, USA

**Keywords:** 3D organoids, esophageal adenocarcinoma, esophageal squamous cell carcinoma, Barrett’s esophagus, microbiome, dysbiosis, barrier function, host–pathogen interactions

## Abstract

The microbiome is an emerging key co-factor in the development of esophageal cancer, the sixth leading cause of cancer death worldwide. However, there is a paucity of data delineating how the microbiome contributes to the pathobiology of the two histological subtypes of esophageal cancer: esophageal squamous cell carcinoma and esophageal adenocarcinoma. This critical knowledge gap is partially due to inadequate modeling of host–microbiome interactions in the etiology of esophageal cancers. Recent advances have enabled progress in this field. Three dimensional (3D) organoids faithfully recapitulate the structure and function of the normal, preneoplastic, and neoplastic epithelia of the esophagus ex vivo and serve as a platform translatable for applications in precision medicine. Elsewhere in the gastrointestinal (GI) tract, the co-culture of 3D organoids with the bacterial microbiome has fostered insight into the pathogenic role of the microbiome in other GI cancers. Herein, we will summarize our current understanding of the relationship between the microbiome and esophageal cancer, discuss 3D organoid models of esophageal homeostasis, review analogous models of host–microbiome interactions in other GI cancers, and advocate for the application of these models to esophageal cancers. Together, we present a promising, novel approach with the potential to ameliorate the burden of esophageal cancer-related morbidity and mortality via improved prevention and therapeutic interventions.

## 1. Introduction

The gastrointestinal (GI) tract harbors a substantial portion of the human microbiome, which plays a critical role in organ development, immunity, nutrition, and maintenance of homeostasis through symbiotic interactions with the host. The gut microbiome influences the pathobiology and therapy response in GI cancers [1,2,3,4]. Since the composition of the microbiome is distinct in each GI organ, the esophageal microbiome may have a unique role in esophageal mucosal homeostasis and pathology [5]. However, little is known about the role of the esophageal microbiome in esophageal pathologies including neoplasia.

Esophageal cancer is the sixth leading cause of cancer death worldwide and the incidence of this disease is expected to increase ~35% by 2030 [6]. Thus, there is an urgent unmet need to characterize the mechanisms promoting esophageal carcinogenesis. The bacterial microbiome is an emerging co-factor in the pathobiology of esophageal cancer and is linked to tumorigenesis and altered treatment response [7,8,9,10]. Despite its incipient role in esophageal malignancies, how the bacterial microbiome contributes to the pathogenesis of esophageal cancer is unclear. This critical knowledge gap is exacerbated by the lack of tractable models of pathogen–host interactions in the esophagus.

Recent advancements in the field of GI oncology include preclinical and translational applications of the three-dimensional (3D) organoid system that recapitulate original tissues from patients and mice to model normal and neoplastic epithelia [11,12,13,14,15]. Genetic and pharmacological manipulations as well as the co-culture of 3D organoids with known pathogens have enabled progress in understanding how these interactions contribute to malignancies elsewhere in the GI tract [15,16,17]. However, these techniques have not been applied in analogous models of esophageal cancer initiation and development.

In this review, we will briefly summarize the relationship between the microbiome and esophageal neoplasia, discuss 3D organoid models of esophageal malignancies, highlight analogous GI models of host–pathogen interactions, and underscore the value of applying such models to esophageal disease.

### 1.1. Esophageal Structure and Function

The esophagus is the elongated, tubular organ that transfers food from the pharynx to the stomach. The stratified squamous epithelium lines the esophageal lumen and consists of proliferating basal cells that undergo post-mitotic terminal differentiation in the suprabasal cell layers. In collaboration with secreted mucin and swallowed saliva, the suprabasal epithelial layer forms a protective barrier against intraluminal contents such as acid reflux from the stomach and the microbiome [18,19]. Several sublayers comprise the stratified squamous epithelium [20]. The outer layer consists of more differentiated suprabasal cells. Beneath the suprabasal cells are the parabasal and basal cells, the latter of which are the putative esophageal stem cells [20,21,22,23]. In humans, the parabasal cells undergo mitosis and function to replenish the epithelium [19,21]. This turnover is rapid, occurring every 4–6 days in mice and every 11 days in humans [24]. Together, the different sublayers of the stratified squamous epithelium collaborate to form the junction complex. This complex includes tight junctions, adherens junctions, and desmosomes, and restricts paracellular mobility of ions, molecules, and microbes [25]. Esophageal epithelial cells can rapidly (<20 min) replenish any disrupted barrier due to their ability to migrate and fill wound margins through the process of restitution [26]. Together, the stratified squamous epithelium is a complex and dynamic tissue that forms a barrier between the esophageal lumen and the underlying tissue. Underscoring its importance, the epithelial barrier is disrupted in esophageal pathologies, including eosinophilic esophagitis, gastroesophageal reflux disease, Barrett’s esophagus, esophageal adenocarcinoma (EAC), and esophageal squamous cell carcinoma (ESCC). Therefore, understanding the molecular features ensuring proper barrier function is critical for unravelling the mechanistic underpinnings of a diverse class of diseases.

### 1.2. Esophageal Squamous Cell Carcinoma

ESCC is the predominant histological subtype of esophageal cancer worldwide, accounting for >90% of all esophageal cancers [27,28]. In recent years, there has been considerable progress documenting the genetic and epigenetic changes promoting ESCC initiation and development of esophageal cancer [29,30,31,32]. Despite these advancements, overall survival remains poor at approximately 20% [33]. Therefore, more work is needed to characterize both the intrinsic and extrinsic factors that promote ESCC tumorigenesis and to translate these findings into actionable therapeutic strategies.

ESCC arises from malignant transformation of proliferating basal cells [20]. Common genetic lesions associated with ESCC are alterations in tumor suppressors *TP53*, *CDKN2A* and oncogenes *PI3KCA*, *EGFR*, *CCND1*, and *SOX2* [31,34,35,36]. Intriguingly, inflammatory signals from the microenvironment are required for full ESCC initiation and development [37]. These results highlight the importance of characterizing both cell extrinsic and cell intrinsic factors in the etiology of ESCC.

Supporting this concept, there are a diverse set of both genetic and environmental risk factors associated with ESCC. Environmental risk factors include alcohol consumption, tobacco use, intake of hot liquids, ingestion of areca nut, and deficiencies in vitamins A, C, and trace elements such as zinc [6]. Besides environmental factors, geographic location is also associated with ESCC development, although it is unclear whether this distribution is the result of common genetic lineages, common environmental factors, or both [38]. One such region is East Asia, where polymorphic mutations to aldehyde dehydrogenase 2 (*ALDH2*) or alcohol dehydrogenase 1B (*ADH1B*) are associated with an increased risk of developing ESCC [6]. In Chinese populations, mutations in phospholipase C are associated with the increased incidence of ESCC [39]. A second hotspot is the “African esophageal cancer corridor,” which includes eastern, central and southern sub-Saharan Africa [38]. Poor oral health has been linked to ESCC in this corridor, highlighting how environmental factors can influence the geographic distribution of this disease [40]. Together, the diversity of intrinsic and extrinsic risk factors results in dramatic heterogeneity in the distribution of ESCC worldwide. Some areas have ~10-fold increase in the number of ESCC cases [6]. Importantly, although many of these risk factors could have important effects on the composition of the microbiome, few studies have considered its impact on the development of ESCC [41]. These studies will be discussed in a later section.

### 1.3. Esophageal Adenocarcinoma

EAC is the predominant histological subtype of esophageal cancer in North America and Western Europe [6]. There has been similar progress in characterizing the molecular features of EAC as in ESCC; however, these findings have not translated into better patient outcomes [6]. Consequently, more work is needed to better characterize the cell intrinsic and extrinsic factors associated with this disease.

Unlike ESCC, the cell-of-origin for EAC remains controversial [20]. EAC frequently arises from Barrett’s esophagus (BE), a form of intestinal metaplasia in which the squamous epithelium is replaced by simple columnar cells that harbor features of intestinal differentiation [42]. BE formation is also dependent on a combination of cell intrinsic and extrinsic factors such as inflammation; however, more work is needed to fully characterize the cell intrinsic and extrinsic factors promoting EAC initiation and development [43,44]. Common risk factors for EAC include both genetic and environmental factors. Caucasian males over 50 are at the highest risk for EAC. In addition, gastroesophageal reflux disease (GERD), high fat diet, and obesity are environmental factors associated with increased risk of EAC. While EAC shares some common genetic lesions with ESCC such as inactivating mutations to *TP53* and *CDKN2A*, EAC’s molecular profile is more similar to gastric cancers [29]. In addition to these genetic lesions, the microbiome has been implicated in BE and EAC development [45]. This involvement will be discussed in further detail in a later section.

### 1.4. The Bacterial Microbiome in Esophageal Health and Disease

The bacterial microbiome is an emerging co-factor in esophageal health and disease (see recent reviews for more comprehensive discussion [41,46,47]). While the esophagus was long believed to be sterile, a growing body of evidence suggests that there is a stable esophageal bacterial microbiome that is heavily influenced by the oral microbiome [48,49,50]. In health, the esophageal microbiome primarily consists of 95 taxa belonging to six phyla, *Firmicutes*, *Bacteroides*, *Actinobacteria*, *Proteobacteria*, *Fusobacteria*, and *Saccharibacteria phylum* (also known as *TM7*) [51,52]. The precise function of the esophageal microbiome is unclear; however, recent evidence suggests that bacteria can directly influence the gene expression profiles of esophageal epithelial cells [50]. The impact of individual bacterial species on esophageal epithelial homeostasis is yet to be explored, in part due to a lack of tractable model systems. More work is needed to annotate the function of the bacterial microbiome both at the global and at the individual species level.

Supporting its role as a co-factor in esophageal health and disease, the microbiome is altered in esophageal neoplasia. Decreases in microbiome diversity are associated with ESD, ESCC, BE, and EAC [53,54,55,56,57]. However, contradictory evidence suggests that in some cases, bacterial diversity is either not altered or increased in esophageal neoplasia [52,55,56,57]. At a more granular level, there are changes to individual genera and species in esophageal neoplasia. For example, *Fusobacteria* are enriched in ESCC [55,58]. Further, enrichment of Gram-negative genera *Campylobacter* and alterations to the *Streptococcus:Prevotella* ratio are a reportedly common event in BE [54,58,59,60]. Finally, decreases in *Veillonella* are have been reported in in EAC [45,57], although further studies contradict this claim. Intriguingly, changes in the oral microbiome may predict the development of esophageal neoplasia. The presence of *Tannerella forsythia* in the oral cavity is associated with increased EAC risk while reduced levels of *Neisseria* and *Streptococcus pneumoniae* are associated with lower EAC risk [61]. Further, an additional study identified 11 bacterial species that are associated with an increased risk of ESD and ESCC [58]. These studies raise the possibility that specific bacterial populations may be driving neoplastic change. However, due to the contradictory and largely descriptive evidence discussed above, more work is needed to determine the functional consequences of altered oral and esophageal microbiomes in esophageal neoplasia.

Few studies have elucidated these consequences by employing co-culture models of the microbiome and 2D esophageal cells [62,63,64,65,66,67]. In co-culture models of *F. nucleatum* and ESCC cell lines, Liu and colleagues determined that *F. nucleatum* confers chemoresistance through the modulation of autophagy [64], a cytoprotective mechanism. Further, co-culture models of *P. gingivalis* and 2D ESCC cell lines determined that *P. gingivalis* promotes tumor invasiveness and stemness through Interleukin (IL-6) signaling [65]. Additional co-culture experiments with human esophageal epithelial cells and various BE-associated *Campylobacter* isolates revealed that TNFAIP2, CXCL2, ICAM1, and MANBA transcripts are significantly upregulated following exposure to these bacteria [66]. At the pathway level, several inflammatory pathways such as cytokine–cytokine receptor interaction, tumor necrosis factor (TNF) signaling, and IL-17 signaling were upregulated following *Campylobacter concisus* (*C. concisus*) exposure. Exposure to *Campylobacter rectus* also resulted in TNF and IL-17 signaling upregulation, as well as the upregulation of “transcriptional misregulation in cancer.” Consistently, the co-culture of *C. concisus* and BE cell lines results in increased transcription of TNF-α and Il-18 [67]. Together, these studies highlight how co-culture models can provide clinically actionable insight into the pathological role of the microbiome in ESCC.

Ultimately, the esophagus harbors a stable bacterial microbiome that is altered in esophageal neoplasia. Critically, whether these alterations have a causal role in promoting esophageal neoplasia is unclear. Few studies have addressed the functional consequences of alterations to these microbiomes on the identity and behavior of the underlying esophageal squamous epithelium. Such studies have utilized co-culture models of specific bacteria and esophageal epithelial cells in monolayer. While valuable, such models fail to recapitulate the dynamic esophageal squamous epithelium (Section 1.1) and therefore offer an incomplete understanding of the impact of the microbiome on the underlying tissue. Faithful characterization of this impact will isolate the salient changes in these microbiomes as well as reveal novel therapeutic strategies to more effectively treat two devastating diseases.

### 1.5. The 3D Esophageal Organoid System

Organoids faithfully recapitulate the dynamic esophageal epithelium (Section 1.1) and therefore are a powerful ex vivo tool for modelling esophageal homeostasis and disease [68,69,70]. Esophageal 3D organoids recapitulate normal epithelial renewal, differentiation, and proliferation [69,70]. These organoids can also be used to study disease-specific alterations in response to a variety of pathogenic stimuli [71]. Further, 3D organoids are amenable to genetic and pharmacological manipulation [72]. Ultimately, 3D organoids are a tractable model system that recapitulates many of salient features of normal esophageal homeostasis and a variety of esophageal maladies.

Organoids are cell-culture-based models that can be formed from adult stem cells (ASCs) derived from proliferative basal cells in the esophagus or from induced pluripotent stem cells (iPSCs) [71,73,74]. For ASC-based models, single cells are dissociated and plated into an extracellular matrix (ECM)-based hydrogel that simulates the basement membrane, such as Matrigel (Corning, USA), which is a solubilized basement membrane extracted from Engelbreth–Holm–Swarm mouse sarcomas. This mixture contains laminin, collagen IV, heparan sulfate proteoglycans, entactin/nidogen, and several growth factors. Currently, other ECM mimetics are under development [75]. In Matrigel, esophageal 3D organoids form rapidly (<14 days) from a single cell, self-organizing into a spherical structure that recapitulates the proliferation–differentiation gradient of the stratefied squamous epithelium, with the proliferative basal cells on the outer layer and the more differentiated cells towards the center [71]. In addition to Matrigel, 3D organoids are cultured in liquid media, and histologically distinct esophageal tissues require different media formulations [68,69,76]. We have demonstrated that keratinocyte serum free supplemented with calcium (KSFMc)-based media can be used to successfully generate organoids from normal (100% success rate) but not SCC tissue [70]. Advanced Dulbecco’s Modified Eagle’s Medium (aDMEM)-based culture methods have higher success rates growing SCC tissue (71.4%) but reduced success growing organoids from normal cells (66.7%) [69]. EAC organoids grow (80%) in aDMEM-based media that has been supplemented with Wnt3a and increased levels of EGF [76]. Ultimately, media formulation is an important consideration in 3D organoid culture and highlights how cell extrinsic factors greatly influence the recapitulation of different histological subtypes of esophageal health and disease.

In addition to ASC or cell line-based organoids, iPSC-based esophageal organoid models have recently been developed [77,78]. Esophageal progenitor cells (EPCs) can be generated through sequential specification of human pluripotent stem cells. EPCs can then be differentiated to recapitulate the normal development of the esophageal squamous epithelium. This system is generally used to study the development of the fetal esophagus, but organoids take several weeks to form, which is significantly slower than the formation of organoids from ASCs or cell lines. Together, there are several methods for forming 3D esophageal organoids, each with their strengths and weaknesses.

These 3D organoids are also used in co-culture with other cell types. Recent efforts have focused on expanding these co-culture models to incorporate elements of the immune system to enhance the physiological relevance of organoid models as well as predict response to immunotherapy [79]. Future studies such as the work proposed herein should expand co-culture models to include other cell types and organisms to more effectively capture the physiological processes underlying human health and disease.

Ultimately, 3D organoids are physiologically relevant models that retain the structure of their tissue of origin, incorporate cell-extrinsic signals, grow rapidly from a variety of different sources, and are commonly used in co-culture with other cell types. Together, these features position 3D organoid models as an ideal platform for examining host–pathogen interactions in the co-culture of the microbiome and esophageal tissue.

## 2. Organoid and Microbiome Co-Culture Models of GI Cancer-Relevant Processes

Elsewhere in the GI tract, co-culture models of 3D organoids and the gut microbiome have enabled the faithful characterization of the consequences of microbe–epithelial interactions [17,80,81]. These studies have focused on the effect of specific bacteria or bacterial metabolites on cancer-relevant processes in the gastric or intestinal epithelium, including proliferation, viability, inflammatory signaling, immunogenicity, genomic stability, and cell fate determination (Table 1). To date, most studies have focused on the effect of *Helicobacter pylori*, a causative agent of gastric cancer, on gastric 3D organoids [82]. However, there is growing interest in modeling the interactions of the GI epithelia and other bacterial species using 3D organoids. We will highlight the GI cancer-relevant studies below and discuss how the 3D organoid system facilitates research in this clinically relevant and incompletely understood field.

### 2.1. Microbiome and Epithelial Cell Proliferation

Unconstrained proliferation is a common feature of cancer cells. Recent studies have leveraged the 3D organoid platform to better characterize the influence of the microbiome on epithelial cell growth and have identified several different species that promote the proliferation of both gastric and intestinal epithelial organoids (Table 1). An early study determined that microinjection of *H. pylori* into the lumen of human gastric organoids results in increased epithelial proliferation through c-Met signaling [83]. A similar study corroborated these findings, demonstrating that *H. pylori* microinjection into the lumen of murine-derived gastric organoids induced proliferation in a CagA- and β-catenin-dependent manner [84]. Further, *H. pylori* resulted in the mislocalization of claudin-7, a tight junction protein required to maintain mucosal epithelial integrity [84]. Further evidence from 3D organoid models suggests that *H. pylori* infection results in both increased proliferation of both patient- and murine-derived gastric organoids and increased epithelial–mesenchymal transition in a CD44-dependent manner [85]. Pretreatment of patient-derived organoids with a CD44 peptide inhibitor resulted in the loss of epithelial proliferation following exposure to *H. pylori*, demonstrating how findings from 3D co-culture models can reveal potential clinic targets for the treatment of microbiome-associated gastric cancers. Together, these studies demonstrate how 3D organoids can be utilized to characterize the molecular consequences of cancer-relevant microbe–epithelial interactions. Beyond *H. pylori* infection, recent evidence suggests that commensal microbiome metabolites can greatly influence the tumorgenicity and proliferative capacity of transformed epithelial tissue [86]. Intestinal tumor organoids derived from mice harboring oncogenic p53 mutations exhibit normal and balanced growth and differentiation in the absence of the microbiome through the disruption of the WNT pathway. However, treatment of these organoids with the bacterial metabolite gallic acid was sufficient to restore T-cell factor-mediated WNT signaling, increase organoid proliferative capacity, and result in a loss of organoid differentiation consistent with transformation. Removal of gallic acid from the culture medium reversed the transformed phenotype, highlighting the plasticity of these cells and presenting the intriguing possibility that modulation of the gut microbiota may be a potential therapeutic avenue for p53-mutated intestinal cancers. Highlighting the value of the 3D organoid system, the authors performed a coarse screen of the effect of many differential bacterial metabolites on intestinal tumor organoid growth and proliferation. This screen was possible because organoids capture the physiology of the original tissue and are easily treated and tracked. Ultimately, 3D organoids facilitated the discovery of a novel and highly cancer-relevant phenotype. In other contexts, 3D organoids have been utilized to demonstrate that bacterial products result in reduced proliferation and increased stem cell death. Treatment of murine intestinal crypt organoids with *E. coli*-derived endotoxin lipopolysaccharide (LPS) results in increased levels of the apoptotic marker cleaved caspase 3 and decreased levels of the proliferation marker PCNA [87]. LPS stimulation had no effect on Toll-like receptor 4 (TLR4) knockout mice. A similar study corroborated these results, demonstrating that LPS stimulation of murine intestinal organoids results in decreased proliferation, increased necroptosis (a programmed form of inflammatory cell death) of stem cells, and increased cell differentiation through a TLR4-dependent program [88]. This study is an elegant example of how the 3D organoid platform can be utilized to identify the molecular mechanisms and consequences of microbe–epithelial interactions. The authors isolated a crypt-specific core microbiota (CSCM) and hypothesized that this bacterial population affects epithelial generation. The authors first determined that the CSCM affected epithelial proliferation and survival in mice, and then employed the 3D organoid system to identify the salient molecular processes driving this change. The authors incubated organoids with sonicates and with purified LPS from four representative CSCM species (*S. maltophilia*, *A modestus*, *A. radioresistens*, and *D. tsuruhatensis*) and measured proliferation, death, and differentiation of epithelial cells. The authors determined that, while LPS from all CSCM species resulted in decreased organoid maturation, LPS from *S. maltophilia* specifically induced epithelial cell differentiation and RIPK3-dependent necroptosis of intestinal stem cells. The use of 3D organoids facilitated these studies by providing a physiologically-relevant platform to measure intestinal epithelial homeostasis using short (7 day) cultures that were easily scaled to include a variety of different bacterial byproducts. Together, these studies demonstrate that 3D GI organoids are a valuable platform for identifying the molecular mechanisms regulating epithelial proliferation and survival.

### 2.2. Microbiome and Inflammation and Immunity

Inflammation is an enabling characteristic of cancer [89]. Recent studies have demonstrated that the co-culture of GI organoids and common GI microbes results in a strong inflammatory response (Table 1). Microinjection of *H. pylori* into the lumen of gastric organoids results in a rapid (2 h) increase in NF-κB -regulated proinflammatory genes, including IL-8 [90]. Contradicting data from 2D cell lines, IL-8 expression in gastric organoids did not depend on bacterial cytotoxicity-associated gene pathogenicity island (*cag*PAI) [90,91,92]. These data highlight how experiments performed in 3D organoids and 2D cell lines can produce different results. Organoid co-culture models have also revealed that the pro-inflammatory response to commensal bacterial metabolites can promote epithelial homeostasis. Co-culture of intestinal organoids and the commensal bacteria *Lactobacillus reuteri* (*L. reuteri*) D8 revealed that D8 metabolites stimulate IL-22 expression following intestinal injury, which accelerates epithelial proliferation and promotes barrier integrity [93]. Consistent with these data, microinjection of nonpathogenic *E. coli* into the lumen of intestinal organoids results in increased secretion of IL-6 and IL-8, a transient increase in proliferation, and improved epithelial barrier function [94]. These experiments demonstrate how organoid co-culture contextualizes the effects of the microbiome on the GI epithelium by faithfully recapitulating epithelial barrier function. Avoiding immune destruction is an emerging hallmark of cancer [89]. Recent evidence from microbiome and organoid co-culture has demonstrated that the microbiome can promote immune evasion (Table 1). A co-culture model of *H. pylori* infection in patient-derived organoids and autologous patient cytotoxic T lymphocytes and dendritic cells (DCs) revealed that *H. pylori* induces programmed death-ligand 1 (PD-L1) expression through the Shh signaling pathway [95]. PD-L1 upregulation was rapid (within 48 h) and promoted epithelial cell survival. Treatment with an inhibitor of PD-L1 or programmed cell death protein 1 (PD-1) resulted in epithelial cell death, indicating that *H. pylori*-associated gastric tumors may be susceptible to immunotherapy. An additional study examined the co-culture of patient derived gastric organoids, luminally-microinjected *H. pylori*, and human monocyte-derived dendritic cells [96]. The authors demonstrated that *H. pylori* infection resulted in the recruitment of DCs to the gastric epithelia following the production of multiple chemokines, including CXCL2, CXCL16, CXCL17, and CCL20. These results indicate that the gastric epithelium can recruit DCs for immunosurveillance following *H. pylori* infection. Together, these data highlight how organoid co-culture models can be used to characterize important and targetable mechanisms underlying microbiome-associated GI cancers.

### 2.3. Microbiome and Mutagenesis

Loss of genomic stability is an enabling characteristic of cancer [89]. How bacteria may promote mutagenesis is unclear, in part due to the challenges performing long term co-culture experiments with human epithelial cells and microbiomes. To address this knowledge gap, a recent study performed a long term (5 months) co-culture through repeated microinjection of pathogenic *polyketide synthetase* (*pks*) + *E. coli* into healthy human intestinal organoids [97]. The authors demonstrated that *pks* + *E. coli* generate DNA damage and a distinct mutational signature that is commonly identified in colorectal cancer. Further, short-term infection of primary murine colon organoids with *pks* + *E. coli* results in phenotypes consistent with malignant transformation, including chromosomal aberrations, increased mutational burden, enhanced proliferation, and impaired differentiation [98]. Together, these findings leverage the 3D organoid platform to suggest that a pathogenic bacteria strain has a causal role in GI cancer transformation.

## 3. Discussion and Future Directions

### 3.1. Strengths and Weaknesses of the 3D Organoid-Microbiome Co-Culture Models

3D organoids provide an intriguing platform for the study of epithelial–microbiome interactions for a variety of reasons. These organoids can be rapidly generated (<14 days) and passaged multiple times [71]. Additionally, the equipment and reagents required to culture 3D organoids are available in a modern molecular biology laboratory that performs 2D tissue culture [71]. Further, 3D organoids recapitulate the dynamic proliferation-differentiation gradient of the esophageal mucosa and are embedded in Matrigel, which simulates the basement matrix [71]. Therefore, this system is more physiologically relevant than 2D cell culture. Further, organoids can be established from patient samples or from isogenic mouse models. This versatility enables organoids to be used as a platform for personalized medicine or for targeted interrogation of the interaction of specific genes with the microenvironment [71]. Building on this versatility, 3D organoids are amenable to CRISPR-mediated or RNA interference (RNAi)-mediated genomic engineering and can be used for high-throughput screening in the presence of bacteria or bacterial metabolites added to the cell culture media [68]. Ultimately, 3D organoids represent a powerful tool for modeling epithelial–bacterial microbiome interactions in a physiologically relevant way.

However, there are limitations to 3D organoids as platforms for studying epithelial–microbiome interactions. There is a significant learning curve for generating 3D organoids from single cells [99]. Further, maintaining the oxygen gradient and/or anaerobic conditions required for the cultivation of specific aerobic/anaerobic bacterial species is challenging in the setting of tissue culture [81,99]. Additionally, microinjection of bacterial species into the organoid lumen is not well-suited to high-throughput screening. Further, the cell of origin for EAC and BE is controversial and may not be of esophageal origin, so co-culturing normal esophageal organoids with potentially pathogenic bacteria may be exploring early neoplastic changes in the wrong lineage [20]. Finally, reductionist approaches of co-culturing a single or a select few bacterial species with 3D organoids may occlude a common function of commensal bacteria: preventing the colonization of pathogenic bacteria [100]. Therefore, while the co-culture of 3D organoids and the bacterial microbiome is a promising and novel approach for studying host–microbiome interactions in esophageal neoplasia, there are limitations to this model system.

### 3.2. Utilizing 3D Organoid and Microbiome Co-Culture for the Study of Esophageal Health and Disease

**Figure 1:**
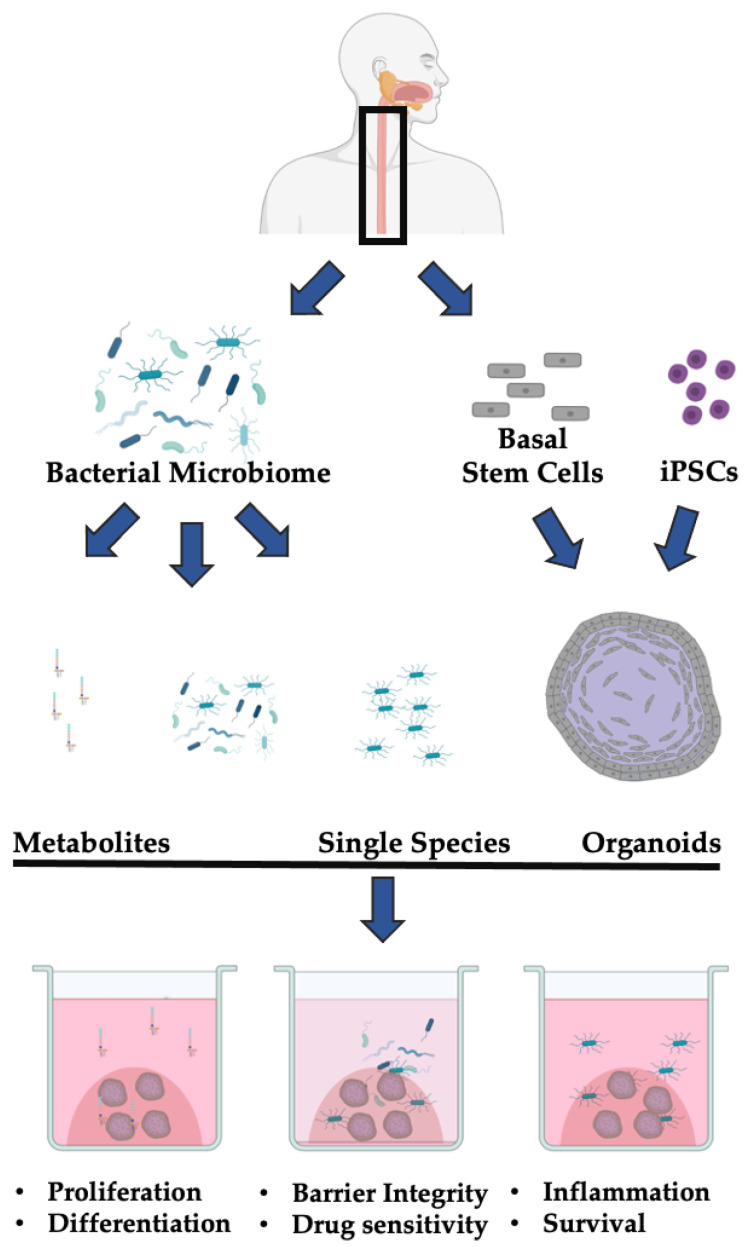
Leveraging 3D organoids to study host–microbiome interactions in esophageal cancers. Patient-derived esophageal tissue can be dissociated into single cells and used to generate organoids. In parallel, the host bacterial microbiome can be isolated and specific bacteria species or products can be cultured or purified, respectively. These cultures or products can then be combined with patient-derived organoids. The ascribed endpoints can be used to assess the effect of the microbiome on the salient biological features of the esophageal stratified epithelium. Created with BioRender.com (accessed on 15 October 2021).

Several studies have indicated that there are stable, esophagus-specific microbial communities primarily composed of *Firmicutes*, *Bacteroides*, *Actinobacteria*, *Proteobacteria*, *Fusobacteria*, and *TM7* [51,52]. Whether these communities affect epithelial homeostasis is unclear. Measuring proliferation, survival, differentiation, and barrier integrity of normal esophageal organoids in co-culture models could provide insight into molecular consequences of these common host–microbe interactions (Figure 1). Further experiments should address the molecular mechanisms of any measured alteration. These experiments have been performed successfully in GI organoids [93,94]. Ultimately, these experiments would address a longstanding question about the role of the bacteria microbiome in esophageal homeostasis. Insights gained from these experiments can then be applied to better understand potentially pathogenic alterations to the microbiome in esophageal neoplasia.

This approach can be applied to study the direct impact of the microbiome on esophageal neoplasia. Several specific bacterial phyla or species have been implicated as risk factors for ESD and ESCC progression, including increased levels of *Fusobacteria* and decreased levels of *Actinobacteria* [55,59]. Characterizing the effect of these and other phyla or species derived from normal, dysplastic, or ESCC tissue, as discussed in Section 2.2 and Section 2.3 on organoids, could provide insight into a potential role for bacteria in the pathobiology of ESCC initiation and development. Similar studies can address the effect of common microbial alterations in EAC and BE. Specifically, the co-culture of 3D organoids from BE or EAC patients with bacteria from the *Campylobacter* genera would enable insight into the mechanistic effects of a common alteration associated with BE. Further, altering the *Streptococcus:Prevotella* ratio in co-culture models of BE organoids could identify the molecular changes that accompany a common microbial alteration [62,63]. Finally, co-culture of EAC-derived organoids with *Veillonella* could provide evidence that a controversial alteration is or is not contributing to EAC pathobiology [57,62]. Each proposed experiment would clarify the consequences of common microbial alterations in esophageal neoplasia.

Co-culturing 3D organoids and the esophageal or oral microbiome may also increase the efficacy of this model as a platform for personalized medicine. Furthermore, 2D co-culture models have been used to demonstrate that elements of the microbiome modulate therapy response [101,102]. There is growing interest in leveraging patient-derived organoids to predict patient response to therapeutics [68]. Future studies should address whether the inclusion of the microbiome from patients increases the predictive potential of this platform.

This co-culture model can be employed to identify the relationship between other microbiome components and esophageal neoplasia. For example, the viral microbiome has a controversial relationship with esophageal cancers. Notably, whether human papilloma virus (HPV) can cause esophageal neoplasia is unclear [103]. HPV is known to cause a variety of squamous cell carcinomas, including some forms of head and neck squamous cell carcinomas [104]. Transfection of HPV E6 and E7 resulted in malignant transformation of 2D fetal esophageal cells; however, this change occurred after 85 passages in a tissue culture flask and therefore may not be physiologically relevant [105]. Recent studies have demonstrated that organoids derived from upper aerodigestive squamous cells can be productively infected with HPV [106]. Future studies should transduce normal esophageal organoids with HPV and measure differentiation, proliferation, barrier integrity, and replicative immortality. Other elements of the viral microbiome such as bacteriophages are also associated with esophageal carcinoma. A recent study comparing the bacteriophage communities of healthy, BE, and EAC patients identified differences in bacteriophage composition between the three groups and determined that genes related to bacterial exotoxin and virulence factors such as LPS biosynthesis were more abundant in rare phages in BE and EAC [107]. The co-culture of esophageal organoids and bacteriophages alone or with components of the bacterial microbiome could better recapitulate host–microbiome interactions to unravel the relationship between bacteriophages and esophageal carcinogenesis. Finally, the fungal microbiome has been implicated in esophageal carcinogenesis, although more work is needed to determine if certain infections have a causative role in promoting malignant transformation [108]. In particular, members of the *Candida* genus (*C. albicans* and *C. glabrata*) are detected in more than half of EAC samples and are also common in ESCC [109,110]. Patients with chronic mucocutaneous candidiasis developed young-onset ESCC in the absence of other known risk factors for esophageal carcinogenesis [110,111,112]. Further, *C. albicans* produces the carcinogens acetaldehyde and benzylmethylnitrosamine [113,114]. Despite this evidence, a causative link between the fungal microbiome and esophageal neoplasia has not been established. The co-culture of 3D esophageal organoids and the fungal microbiome, especially *C. albicans*, could help establish such a link. These experiments could provide more physiologically-relevant insights into the pathologic relationship of a common microbiome component and the esophageal squamous epithelium.

Ultimately, the microbiome is an emerging co-factor in esophageal health and disease. While many studies have documented common components of normal and transformed esophageal epithelial cells, the current understanding of the role of the bacterial microbiome in esophageal homeostasis remains largely descriptive. The co-culture of 3D organoids with both individual bacterial species or the bacterial microbiome isolated from patients will enable functional annotation of these changes (Figure 1).

## 4. Conclusions

The microbiome is an emergent co-factor in the pathobiology of esophageal neoplasia [41]. Within the past two decades, several studies have determined that the composition of the esophageal or oral microbiome is altered in ESCC and EAC as well as their respective precursor lesions ESD and BE. These alterations may have both prognostic and therapeutic value; however, more work is needed to characterize the functional consequences of these changes. The 3D organoid model system represents a powerful tool for capturing the physiology of the normal or neoplastic esophagus. These 3D organoids are easily manipulatable, require little patient material, and are amenable to medium- or high-throughput screening. While no studies have yet leveraged the 3D organoid system to characterize the functional consequences of microbiome alterations in esophageal neoplasia, this system has been applied to other cancer types. Elsewhere in the GI tract, co-culture models of gastric or intestinal 3D organoids have enabled mechanistic insights into how the bacterial microbiome can promote cancer-specific processes such as proliferation, inflammation, immune escape, and mutagenicity. These insights have provided potential therapeutic targets. Therefore, there is growing interest in applying 3D organoid technology to unravel the mechanistic consequences of epithelial–bacterial microbiome interactions in esophageal neoplasia. Additionally, 3D organoid esophageal organoids can be used to identify the functional consequences of epithelial interactions with other elements of the microbiome, including viruses such as HPV. Further, by expanding co-culture models of esophageal organoids with the microbiome and with other stromal or immune cell elements, researchers can better recapitulate the native environment of the human esophagus. This platform would be ideal for personalized medicine. Ultimately, the co-culture of esophageal organoids and the bacterial microbiome is an untapped platform with the potential to provide actionable insight into the pathobiology of a leading cause of cancer worldwide.

## Figures and Tables

**Table 1:** Organoid and microbiome co-culture models of GI cancer-relevant processes.

Tissue	Microbe	Classification	Product	Model	Host	Cancer-Associated Phenotype	Reference
Gastric	*H. pylori*	Pathogenic	Whole bacteria	Luminal microinjection	Human	Increased PD-L1 expression, increased survival	[77]
						Increased inflammatory cytokine production (CXCL2, CXCL16, CXCL17, and CCL20), DC recruitment	[78]
						Increased proliferation through c-Met signaling	[79]
						Increased inflammatory cytokine production through the NF-κB pathway	[80]
					Mouse	Increased proliferation through β-catenin signaling, mislocalization of Claudin-7	[81]
					Human; Mouse	Increased CD44-dependent proliferation and EMT	[82]
Intestinal	*pks + E. coli*	Pathogenic	Whole bacteria	Luminal microinjection	Human	Increased DNA damage and mutational burden	[83]
					Mouse	Increased proliferation, decreased differentiation, increased chromosomal alterations, increased DNA mutational burden	[84]
	*E. coli*	Commensal	Whole bacteria	Luminal microinjection	Human	Increased proliferation (transient), enhanced barrier integrity through IL-6 and IL-8 signaling	[85]
			LPS	Supplemented into media	Mouse	Decreased proliferation, increased apoptosis through TLR4 signaling	[86]
	*Acinetobacter*, *Stenotrophomonas*, and *Delftia* genera	Commensal	LPS	Supplemented into media	Mouse	Decreased proliferation, increased necroptosis, increased differentiation through TLR4 signaling	[87]
	*L. reuteri* D8	Commensal	Whole bacteria, indole-3-aldehyde	Supplemented into media	Mouse	Increased proliferation, enhanced barrier integrity through IL-22 signaling	[88]
	Common commensal metabolites	Commensal	Gallic acid	Supplemented into media	Mouse	Increased WNT signaling, Increased proliferation, decreased differentiation in mutant p53 epithelial cells	[89]

## Data Availability

Not applicable.
